# Different competing risks models applied to data from the Australian Orthopaedic Association National Joint Replacement Registry

**DOI:** 10.3109/17453674.2011.618918

**Published:** 2011-11-24

**Authors:** Marianne H Gillam, Amy Salter, Philip Ryan, Stephen E Graves

**Affiliations:** ^1^School of Population Health and Clinical Practice; ^2^Data Management and Analysis Centre, Discipline of Public Health, University of Adelaide; ^3^Australian Orthopaedic Association National Joint Replacement Registry, Adelaide, Australia

## Abstract

**Purpose:**

Here we describe some available statistical models and illustrate their use for analysis of arthroplasty registry data in the presence of the competing risk of death, when the influence of covariates on the revision rate may be different to the influence on the probability (that is, risk) of the occurrence of revision.

**Patients and methods:**

Records of 12,525 patients aged 75–84 years who had received hemiarthroplasty for fractured neck of femur were obtained from the Australian Orthopaedic Association National Joint Replacement Registry. The covariates whose effects we investigated were: age, sex, type of prosthesis, and type of fixation (cementless or cemented). Extensions of competing risk regression models were implemented, allowing the effects of some covariates to vary with time.

**Results:**

The revision rate was significantly higher for patients with unipolar than bipolar prostheses (HR = 1.38, 95% CI: 1.01–1.89) or with monoblock than bipolar prostheses (HR = 1.45, 95% CI: 1.08–1.94). It was significantly higher for the younger age group (75–79 years) than for the older one (80–84 years) (HR = 1.28, 95% CI: 1.05–1.56) and higher for males than for females (HR = 1.37, 95% CI: 1.09–1.71). The probability of revision, after correction for the competing risk of death, was only significantly higher for unipolar prostheses than for bipolar prostheses, and higher for the younger age group. The effect of fixation type varied with time; initially, there was a higher probability of revision for cementless prostheses than for cemented prostheses, which disappeared after approximately 1.5 years.

**Interpretation:**

When accounting for the competing risk of death, the covariates type of prosthesis and sex influenced the rate of revision differently to the probability of revision. We advocate the use of appropriate analysis tools in the presence of competing risks and when covariates have time-dependent effects.

Arthroplasty registry data are traditionally analyzed with survival methods. The outcome of interest is the time from the primary procedure until revision of the prosthesis. The revision procedure is performed when the prosthesis fails and the time to revision is a crude measure of the success of the arthroplasty.

Competing risk analysis is a sub-discipline of survival analysis. It is relevant where there is more than one outcome of interest, each competing with the occurrence of the other(s). Applications of these methods have become more prevalent in some areas of medical research (Evans et al. 2010); however, they are still infrequently used in orthopedic research. An example of a competing risk event in arthroplasty registry data is death. It is competing because the death of the patient precludes a later revision.

We have previously reported on why one of the standard methods in survival analysis, the Kaplan-Meier method, is not the most appropriate method to estimate the probability of revision in a situation where there is a competing risk such as death (Gillam et al. 2010). When the incidence of death is high, the Kaplan-Meier method may substantially overestimate the probability of revision. Furthermore, if there is also a different incidence of death between treatment groups, the degree of overestimation may be larger for some treatment groups than for others, possibly leading to wrong conclusions about treatment effects. This may occur, for example, due to a selection bias where one treatment is preferred for frail patients with low life expectancy to another for healthy patients with high life expectancy. The reason that the probabilities of revision may be overestimated with the Kaplan-Meier method in the presence of competing risks is because a key methodological assumption in the method is violated—in that not all patients considered at risk of revision in the survival function have the same risk of having a revision (since some of them have died). Instead of using the Kaplan-Meier method in competing risks analysis, a measure of the failure function called the cumulative incidence function (CIF)—which takes into account the competing risk of death—should be employed when estimating the absolute probability of revision at any given time (Kalbfleisch and Prentice 1980, Schwarzer et al. 2001).

In the analysis of registry data, it is often of interest to obtain estimates of revision rates and probabilities of revision adjusted for the effect of covariates. Regression methods for competing risks analysis are available, but to our knowledge there have been no studies in which these methods have been applied to joint registry data. Thus, in this paper we extend the discussion of competing risk methods to the modeling framework, and apply regression to data from the Australian Orthopaedic Association National Joint Replacement Registry (AOA NJRR). Our objectives are (1) to introduce readers to some of the available statistical models for dealing with competing risks when analyzing arthroplasty data, and (2) to illustrate their use by investigating the effects of covariates on both the hazard rates of revision and the probabilities (risks) of revision in patients who received hip arthroplasty as treatment for fractured neck of femur (FNOF). We also discuss strategies for dealing with non-proportionality of the hazards due to covariates whose effects on the outcome vary with time (time-dependent covariates).

## Background to statistical methods

Two commonly used measures of the outcome of interest in time-to-event data are the hazard rate and the failure function. The hazard rate is the instantaneous rate of an event, for example revision, amongst those still at risk of experiencing revision. One can think of the hazard rate at a specified time as reflecting how fast the risk of revision is changing at that time. The failure function describes the probability of revision up to any given time since the primary procedure.

We begin with a brief background to regression methods for assessing the effects of covariates on the hazard rate; that is, the instantaneous revision rate. This is followed by a description of methods for assessment of the effects of covariates on the actual probability of revision in the presence of competing risks.

## Methods for assessing the effect of covariates on the revision rate

The standard regression method in survival analysis is the Cox proportional hazards (PH) model (Cox 1972). This may be used to estimate the effect of covariates on the hazard rate of the event of interest when, for example, exploring etiological factors associated with an event such as revision. In the non-competing risk situation, estimates of the hazard rates of the event of interest from the Cox PH may also be used to calculate directly the adjusted probability of failure (or survival). The relative effect of a covariate is summarized by a hazard ratio. The Cox PH model is said to be a proportional hazards model because the ratio of the hazard rates for two subjects differing in the values of their covariates is assumed to be constant throughout the study period. A major (often neglected) problem when using the Cox PH model (Aalen 2000, Ranstam and Robertsson 2010), is that the hazard rates may not be proportional. One reason for lack of proportionality is when the effect of a covariate on the hazard rate changes over time. For example, the effect may initially be large and then taper off, as may be seen in the effects of an analgesic for chronic pain. This change in effect with time is not captured by the hazard ratio when it is assumed to be constant, resulting in loss of information and possibly wrong conclusions (Bellera et al. 2010).

When covariates have effects that vary with time, a useful alternative to the Cox PH model is Aalen's additive hazard regression model (Aalen 1980, 1989). The Cox PH model is said to be multiplicative because a unit change in the value of a covariate multiplies the hazard of the event of interest by a constant amount. In Aalen's model, the covariates have an additive effect on the hazard rate. The model easily incorporates covariates with time-dependent effects. The effects of covariates cannot be summarized in a single measure, but the estimated cumulative regression coefficient functions can be visualized in graphs.

Scheike and Zhang (2002) have developed an extension of the Cox PH model, called the Cox-Aalen model, which combines the multiplicative Cox PH model and the additive Aalen model. The advantage of this model is its flexibility in allowing covariates with constant effects to be summarized with hazard ratios, and covariates with time-dependent effects to be presented in graphs, thus displaying how effects on the hazards vary with time.

## Methods for assessing the effect on the probability of revision

In the presence of competing risks, the Cox PH model is a valid method for estimation of each cause-specific hazard rate. (In arthroplasty registry data, the cause-specific hazard rate of interest will typically be the rate of revision at a given time; the other rate of interest will be that of death). However, in contrast to standard survival analysis with only one cause of failure, the influence of a covariate on the cause-specific hazard rate and on the probability of failure (which is estimated by the CIF) for that cause may be quite different. For example, an increase in a covariate value may lead to an increase in the hazard rate for an event, but the actual probability of occurrence of that event may be unaltered or may even decrease. This is because the CIF depends on all the cause-specific hazard rates, not just on the hazard rate for the specific cause of interest. In order to estimate the absolute probabilities of an event without first having to model all the cause-specific hazard rates, methods have been developed to model the effects of covariates where the estimates can be used to calculate the CIF directly (Zhang et al. 2008). The model most often used is the subdistribution hazard model of Fine and Gray (1999). It is similar to the Cox PH model in that the baseline subdistribution hazard rate is left unspecified and the subdistribution hazard rates are assumed to be proportional. Hence, as for the Cox PH model, the validity of the estimates obtained from the Fine and Gray model depend on the effects of the covariate(s) remaining constant, that is, being independent of time.

For competing risks analysis with covariates whose effects vary with time, Scheike and Zhang (2008) have developed models based on a binomial regression approach. In one of these models, the covariates are categorized into covariates with multiplicative constant and multiplicative time-dependent effects. When all covariates are modeled with only multiplicative constant effects, the model is equivalent to the Fine and Gray subdistribution model. (To avoid confusion, we will term the model that includes both constant and time-dependent covariates a “modified Fine and Gray model”). In another of these models, the so-called “semi-parametric additive model”, covariates may have additive constant or additive time-dependent effects.

## Patients and methods

Data on 12,525 patients aged 75–84 years who received hemiarthroplasty for fractured neck of femur in the 7-year period from January 1, 2002 to December 31, 2008 were obtained from the Australian Orthopaedic Association National Joint Replacement Registry (AOA NJRR).

The covariates used in our models were: age, sex, type of prosthesis (monoblock, unipolar or bipolar) and type of fixation of the prosthesis (cementless or cemented). For descriptive purposes, the variable “age” was dichotomized as patients aged 75–79 years and those aged 80–84 years. For all analyses, we excluded patients who had bilateral procedures. Revisions were defined as reoperations of primary hip replacements and involved the insertion, removal, and/or replacement of one or more components used in the primary procedure.

### Statistical analyses

The outcome of interest was “time to first revision”, being the time interval between the date of the primary arthroplasty and the date of first revision. The competing risk event was death. Observations were right-censored on December 31, 2008 if neither revision nor death had yet occurred.

We estimated the probability of revision and of death over the study time for each covariate separately using cumulative incidence functions, and tested for differences in the CIF among levels of each covariate using Gray's test (Gray 1988).

The joint effects of the covariates on the cause-specific hazard of revision were initially explored by fitting a Cox PH model, and the proportional hazards assumption was tested by including time interactions with each covariate. Similarly, a Fine and Gray model was fitted to assess how the covariates influenced the absolute probability of revision, and time interactions were examined. Fixation type (cementless or cemented) and age group in both models showed non-constant effects, and in order to evaluate this further, a Cox-Aalen and a modified Fine and Gray model were fitted. Goodness of fit and time-varying effects for all covariates were evaluated with Schoenfeld and Cox-Snell residuals and using methods described by Scheike and Zhang (2008). Subsequently, models were chosen where the effect of fixation type (cementless or cemented) was included as a time-dependent component and other covariates were modeled as constant. The dichotomized covariate age group showed an effect on rate and probability of revision that varied with time; however, we chose to model it as constant, as the effect on the other covariates was negligible and the main interest was how the effect of fixation varied with time.

The modified Fine and Gray model was further evaluated by comparing it with a semi-parametric additive model with both constant and time-dependent covariate effects. To illustrate how the additive effect on the probability of revision of type of fixation varied with time, we prepared a graph based on the semi-parametric additive model showing the difference in the cumulative subdistribution hazard rates of revision for cementless vs. cemented fixation of the prostheses. The slope of the line indicates how the additive effect on the subdistribution hazard varies with time, or not. For example, if the slope of the curve can be represented by a straight line, this would indicate a constant effect. A positive slope indicates an increasing effect with increasing covariate value and a zero slope indicates no effect corresponding to that covariate. Since the CIF can be calculated directly from these estimates, the changes in the slope also indicate how the difference in absolute probability (risk) of revision between the two groups is changing.

In order to illustrate the bias in the predictions of the probability of revision when not accounting for the competing risks, predictions of the failure function based on both the Cox-Aalen model and the modified Fine and Gray model for two different categories of patients were estimated and compared graphically. The two categories were chosen as best and worst case with respect to revision rates as indicated by the Cox-Aalen model.

### Software

For the calculations, we used the packages “timereg” (Martinussen and Scheike 2006) and “cmprsk” (Gray 2010) from the software environment “R” (R Development Core Team 2011).

## Results

Summary statistics for the independent variables (age, sex, prosthesis type, and fixation) are presented in Table 1.

**Table 1.** Distribution of outcomes by covariate status

Covariate	Censored **^a^**	Revised **^b^**	Deceased	Total
Prosthesis type				
Monoblock	2,348 (40%)	225 (4%)	3,229 (56%)	5,802
Unipolar	2,522 (70%)	98 (3%)	990 (27%)	3,610
Bipolar	1,923 (62%)	73 (2%)	1,117 (36%)	3,113
Age				
75–79 years	2,802 (58%)	180 (4%)	1,881 (39%)	4,863
80–84 years	3,991 (52%)	216 (3%)	3,455 (45%)	7,662
Sex				
Males	1,420 (42%)	114 (3%)	1,849 (55%)	3,383
Females	5,373 (59%)	282 (3%)	3,487 (38%)	9,142
Fixation				
Cemented	4,301 (62%)	146 (2%)	2,490 (36%)	6,937
Cementless	2,492 (45%)	250 (4%)	2,846 (51%)	5,588
Total	6,793 (54%)	396 (3%)	5,336 (43%)	12,525

**^a^** Right-censored due to closure of database for analysis.

**^b^** Simple raw proportion, not allowing for censoring.

**Figure 1.**
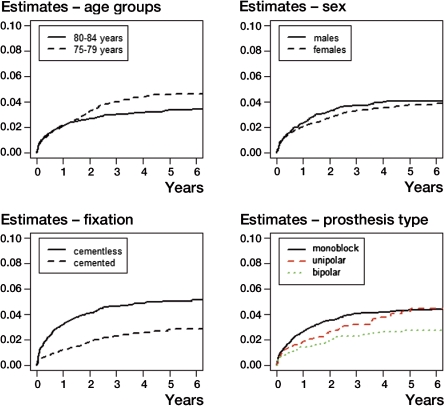
Estimates of CIFs for revision, for each variable.

**Figure 2.**
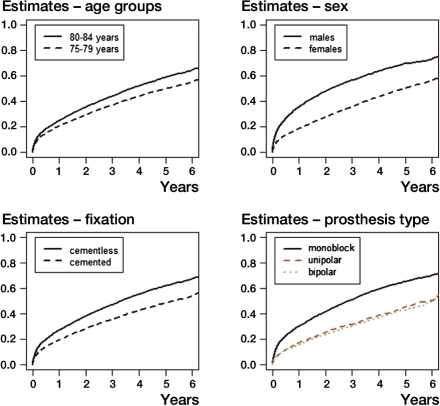
Estimates of CIFs for death, for each variable.

Figure 1 shows the estimated CIFs for revision; that is, the estimated probability of revision at various time points for each covariate. The probability was the same for the two age groups for approximately the first 1.5 years, after which the probability of revision was higher for the younger age group (p = 0.009). There was no evidence of a difference in the probability of revision between the sexes. The CIFs for revision for fixation showed that patients with cementless fixation had the highest probability of revision (p < 0.001). The CIFs for types of prostheses indicated that patients with a bipolar prosthesis had a lower probability of revision than each of the two other types (p < 0.05 for all differences) at each time point, whereas there was no difference in probability between the monoblock and unipolar prostheses. The CIFs of the competing risk of death for each variable are shown in Figure 2. Patients in the older age group, males, patients with monoblock prostheses, and prostheses with cementless fixation had the highest probability of death at each time point (all p < 0.001).

### Joint effects of covariates

The estimates from the Cox-Aalen model and the modified Fine and Gray model, both with fixation type included as time-dependent, are presented in Table 2. The Cox-Aalen model indicated that age group, sex, and monoblock/unipolar vs. bipolar prostheses had a statistically significant effect on the rate of revision. Estimates from the modified Fine and Gray model indicated that only effects of age group and unipolar vs. bipolar prostheses were statistically significant. Patients with unipolar prostheses had a 1.38 times (95% CI: 1.01–1.89; p = 0.04) higher revision rate than patients with bipolar prostheses. The subdistribution hazard ratio (from the modified Fine and Gray model) was increased (subdistribution HR = 1.44, 95% CI: 1.04–1.98; p = 0.03) for the unipolar prosthesis group compared to the bipolar group, reflecting the fact that the probability of revision was also higher for this group (Table 2).

**Table 2.** Estimates of hazard and subdistribution hazard ratios of revision based on a Cox-Aalen model and a modified Fine and Gray model, respectively. The effect of fixation varies with time

Models	Cox-Aalen		Modified Fine and Gray	
	HR (95% CI)	p-value	subHR (95% CI)	p-value
Age: young **^a^** vs. old **^b^**	1.28 (1.05–1.56)	0.01	1.36 (1.10–1.67)	0.004
Male vs. female	1.37 (1.09–1.71)	0.007	1.04 (0.83–1.31)	0.7
Fixation type	–	–	–	–
Monoblock vs. bipolar	1.45 (1.08–1.94)	0.01	1.30 (0.97–1.74)	0.08
Unipolar vs. bipolar	1.38 (1.01–1.89)	0.04	1.44 (1.04–1.98)	0.03
Monoblock vs. unipolar	0.95 (0.74–1.22)	0.7	1.11 (0.85–1.45)	0.5

**^a^** 75–79 years old.

**^b^**  80–84 years old. HR: hazard ratio; subHR: subdistribution hazard ratio.

Although patients with monoblock prostheses had a statistically significantly higher revision rate than patients with bipolar prostheses (HR = 1.45, 95% CI: 1.08–1.94; p = 0.01), there was no statistically significant difference in the subdistribution HR (1.30, 95% CI: 0.97–1.74; p = 0.08), indicating no difference in the probability of revision between these two patient groups. This is probably because at any time, the probability of death was higher for patients with monoblock prostheses than for patients with bipolar prostheses (Figure 2), leaving fewer patients with monoblock prostheses left to experience revision. Similarly, there was a statistically significant difference in revision rate between males and females (HR = 1.37, 95% CI: 1.09–1.71; p = 0.007), but the subdistribution hazard rates of revision between the sexes were not significantly different (subdistribution HR = 1.04; 95% CI: 0.83–1.31; p = 0.7), indicating that there was no difference in the probability of revision. These examples show how the Fine and Gray model adjusts for the competing risk of death and thereby reflects clinical reality.

**Figure 3.**
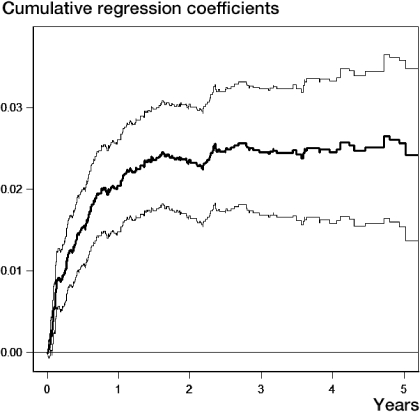
Effect of cementless fixation vs. cemented fixation on the subdistribution hazard of revision with 95% pointwise confidence bands. The slope of the curve indicates the additional probability of revision for cementless fixation in relation to cemented fixation.

The time-dependent effect of cementless fixation vs. cemented fixation from a competing risks semi-parametric additive model is shown in Figure 3. The graph shows the estimated cumulative regression function, and the slope of the curve corresponds to the difference between the subdistribution hazard of cementless and cemented fixation. The slope increased sharply immediately after the primary procedure, then slowed until approximately 1.5 years after which there was no further increase.

**Figure 4.**
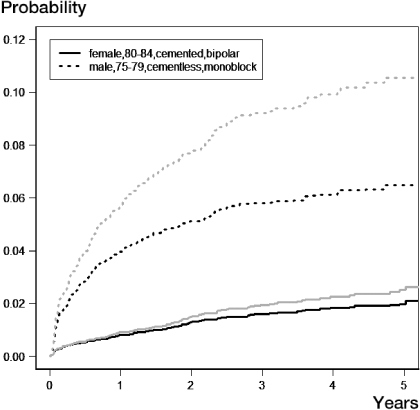
Comparison of predictions of revision based on a Cox-Aalen model (gray) and a modified Fine and Gray model (black). The effect of type of fixation varies with time.

### Predicted probabilities

Figure 4 shows the predicted probability of revision at different time points for two groups of patients using estimates from the modified Fine and Gray model and the Cox-Aalen model (Table 2). Considering first the estimates from the modified Fine and Gray model, the lowest predicted risk is associated with females in the oldest age group receiving a cemented bipolar prosthesis. The probability of revision after 5 years in this group is estimated to be around 2%. For a male patient in the youngest group receiving a cementless monoblock prosthesis, the estimated probability of revision at 5 years is approximately 6%. The predicted risks of revision based on the Cox-Aalen model, which does not account for the competing risk of death, are shown in gray and illustrate that the probability is overestimated in each of the two groups. This is most pronounced for the group with the highest mortality. In this group, the predicted probability of revision after 5 years is more than 10% (compared to 6% for the modified Fine and Gray model).

## Discussion

Adjusting for age, sex, and type of fixation, we found a statistically significantly higher revision rate for patients who received monoblock or unipolar prostheses than for those who received bipolar prostheses. However, the probability of revision, taking the risk of death into account, was only statistically significantly higher for patients with unipolar prostheses compared to those with bipolar prostheses.

The above illustrates important aspects of competing risk analysis. The influence of covariates on the hazard rate may be different to the influence on the actual probability of occurrence of the event. The CIF (or probability) is a function of the hazard rates for all the events. Thus, to gain a full understanding of the results one also has to inspect the incidence of the competing risks for each covariate. If, for example, the revision rate for patients with monoblock prostheses in this study was applied to a healthier population with a lower incidence of death, the probability of revision would probably be higher for that group. Both measures of the outcome are of interest. The effect of covariates on the hazard rate may provide information about etiological associations, while the probability of the occurrence of the event may be of interest with regard to allocation of resources, health planning, and individual risk assessment for patients (Lau et al. 2009).

We found evidence of non-proportionality of the effect of fixation type—that is, the effect on the hazard rate and on the probability of revision varied with time. Patients with cementless prostheses had an early increased probability of revision compared to patients with cemented prostheses, but this effect disappeared after about 1.5 years. Thus, if patients with a cementless prosthesis had not experienced revision within 1.5 years, there was no difference in the probability of revision compared to patients with cemented prostheses, holding other covariates constant.

The Cox PH model is a widely used tool in survival analysis, but the assumption of proportionality is not always checked (Ranstam and Robertsson 2010). There are several ways to handle lack of proportionality, such as stratifying on the covariate, using a weighted average of the hazard ratio, calculating piecewise constant hazard ratios, and time-by-covariate interactions (Schemper et al. 2009). Aalen's additive model (and its extensions that we used in this study) is attractive because of how it enables the effects of covariates that vary with time to be visualized. The model is not as widely used as the Cox PH model, possibly due to lack of awareness about it, as well as lack of generally available statistical software. One example of use in orthopedic research in a non-competing risk situation is in a recent study by Lie et al. (2010), who examined early postoperative mortality after arthroplasty. Also, the additive effect may be different to the multiplicative effect of a covariate. In the present study, both multiplicative effects (not shown) and additive effects of cementless fixation compared to cemented fixation were evident shortly after the primary operation, with the effect diminishing earlier for the multiplicative effect than for the additive effect. As mentioned previously, the estimates from the Aalen model cannot be summarized in single measures. A more commonly used method to handle non-constant hazard ratios in the Cox PH model is to partition the time axis and calculate hazard ratios for specified time intervals where they are constant. This was done in the AOA NJRR Report 2010 (AOANJRR 2010), where the time-dependent effect of fixation type was described by piecewise constant hazard ratios for different hemiarthroplasties, also suggesting an early multiplicative increased effect on revision rate of cementless fixation compared to cemented fixation.

The association between indications for revision and the apparent early increased risk of revision of cementless hemiarthroplasties is of interest. Using data from the Swedish Hip Arthroplasty Registry, Hailer et al. (2010) found an 8-fold increase in the rate of revision due to periprosthetic fractures for cementless stems compared to cemented stems in total hip arthroplasties in the 2 first years after the primary procedure. Further studies of the association between reasons for revision and prosthesis-, patient-, and surgeon-specific factors and how the risk of revision varies with time will be an important addition to future research.

Patients who received monoblock prostheses had higher mortality than patients who received other types. This was probably due to a selection by the surgeons; that is, the frailest patients were given monoblock prostheses. Similarly, there was higher mortality in patients who received cementless fixation than in those who received cemented fixation, and the surgeons may have chosen to give the frailest patients cementless prostheses.

The predicted probability of revision based on the Cox-Aalen model was overestimated compared to predictions based on the modified Fine and Gray model. The overestimation was most pronounced in the group with the highest incidence of the competing risk death. The Cox PH model and its extensions tends to overestimate the predictions of the event of interest in the presence of a competing risk, because the competing risk event leads to a censored observation in the analysis, thus assuming that patients are still at risk of the event (Putter et al. 2007, Wolbers et al. 2009). Hence, in the presence of a high incidence of a competing risk, it is not appropriate to calculate predictions based on estimates of the event of interest only from a Cox PH or Cox-Aalen model.

There is evidence that displaced fractures of the neck of femur in the elderly should in certain circumstances be treated with arthroplasty; however, the type of arthroplasty and type of fixation are unclear (Keating et al. 2006, Frihagen et al. 2010). The results from the present study suggest that cemented bipolar prostheses may have better outcome with respect to revision than other types of hemiarthroplasty. A systematic review (Ahn et al. 2008) comparing cemented prostheses with cementless prostheses found no conclusive difference between the fixation methods for several outcomes (blood loss, operative time, mortality, pain, and revision), but this review did not examine different implant types; nor did it account for competing risks. A recent Cochrane review of randomized controlled trials compared different types of arthroplasties as treatment for hip fractures (Parker et al. 2010). It concluded that there was evidence for better mobility and less pain with cemented arthroplasties than with cementless, no difference between bipolar and unipolar hemiarthroplasties, and better functional outcome for THA compared to hemiarthroplasty. The results with respect to revision were inconclusive. However, a need for more well-conducted randomized trials was noted.

The present study has several limitations. We only examined one measure of the success (or otherwise) of the arthroplasty—time to revision—which is a crude although unambiguous measure. The data were from a registry, and as such there was a limited number of variables recorded. Several covariates that could influence the time to revision such as patient morbidity, surgical technique, surgical waiting lists, etc. were unavailable. Furthermore, it was an observational study based on registry data and the median follow-up time was short (3.4 years estimated with the “reverse Kaplan-Meier” method (Schemper and Smith 1996)). On the other hand, the data were comprehensive since almost 100% of hip arthroplasties performed in Australia are reported to the registry, and they were from a large and unselected population.

Arthroplasty registry data are traditionally analyzed with standard survival methods, that is, the Kaplan-Meier method and the Cox PH method. In the presence of a high incidence of competing risks, competing risks methods should be used in the analysis of the data and care must be taken in interpretation of the results (Lau et al. 2009, Gillam et al. 2010). We are aware of only a small number of orthopedic studies that have used competing risks methods: for example, Maurer et al. (2001), Schwarzer et al. (2001), Doets et al. (2006), Biau et al. (2007), and Fennema and Lubsen (2010). Schwartzer et al. (2001) considered separate Cox models for revision and death in the interpretation of revision rates in a clinical study comparing two different types of femoral stems. The same study material was used by Graw et al. (2009) to examine the properties of two competing risks models. To our knowledge, the present study is the first one to apply competing risks regression methods to joint registry data. One of the reasons why competing risks analysis has not yet been widely used may relate to the lack of user-friendly statistical software to perform the analyses (Zhang et al. 2008). This is now changing, and it is important that researchers in orthopedics and other areas of medicine are aware of competing risks methods and when it is appropriate to use them.
